# Prevalence of Refractive Error Among School Children in Malawi

**DOI:** 10.1155/joph/3313963

**Published:** 2026-03-04

**Authors:** Grace Ogbonna, Phillip Nyambalo, Rosemary Ogbonna, Fidelis Anyika, Ngozika Ezinne, Thokozani Mzumara

**Affiliations:** ^1^ Department of Optometry, Faculty of Health Sciences, Mzuzu University, Mzuzu, Malawi, mzuni.ac.mw; ^2^ Optometry Unit, Abia State Teaching Hospital, Umuahia, Abia, Nigeria; ^3^ Department of Surgery, Imo State University, Owerri, Imo, Nigeria, imsu.edu.ng; ^4^ Optometry Unit, Department of Clinical Surgical Science, University of the West Indies, Saint Augustine, Trinidad and Tobago, uwi.edu; ^5^ Ophthalmology Unit, Department of Curative and Rehabilitation Services, Mzuzu Urban Community Hospital, Mzimba North District Health Office, Mzuzu, Malawi

**Keywords:** corrective lenses, hyperopia school children, Mzuzu City, refractive error, school eye health program

## Abstract

**Background:**

Uncorrected refractive errors are a significant public health concern. Refractive error remains a common cause of vision impairment. Children are vulnerable to uncorrected refractive errors, which can impact their development, education, and social welfare. Hence, it is essential to understand the extent of uncorrected refractive error in different populations to facilitate healthcare planning and the implementation of necessary interventions.

**Aim:**

The study aimed to determine the prevalence of refractive error among school‐going children in five primary schools in Mzuzu City.

**Method:**

This was a cross‐sectional school‐based study conducted among school‐going children in Mzuzu City. A multistage sampling technique was used to recruit 1173 participants. Participants who met the study’s inclusion criteria were refracted under cycloplegia. The following was defined as ≥ 0.5 diopters, while hyperopia was ≥ +1.00 diopters. On the other hand, astigmatism was regarded as 0.50 Dcyl. The data were entered into excel then into SPSS Version 26. Binary logistic regression was used to determine associations. *p* < 0.05 was considered *e* statistically significant.

**Results:**

Among the study population, males and females contributed 586 (49.96%) and 587 (50.04%), respectively. The prevalence of refractive error was 649 (55.3%, CI = 0.5252.0.5828), with a higher incidence in males than that in females. Hyperopia was the most prevalent type of refractive error, occurring in 610 (52.0%) of the study participants. Accordingly, adolescents had 1.445 higher odds of having refractive errors compared with younger children (95% CI = 1.141.1.829, *p* = 0.002).

**Conclusion:**

The high population of students who have refractive errors, without having undergone any form of screening, points to a need for greater efforts at establishing a sustainable school eye health program within the region. Therefore, this study recommends establishing a school eye health program to ensure that every child has the opportunity for visual screening. Furthermore, there is a need to abridge the cost of spectacles through ensuring the provision of readymade spectacles to indigent pupils whose parents may not afford regular spectacles.

## 1. Introduction

Worldwide, refractive error remains a common cause of vision impairment [1]. Globally, it is estimated that over 1 billion people are affected by uncorrected refractive error [2]. Underdiagnosed and largely uncorrected refractive error remains a significant challenge in both low‐ and high‐income countries [1, 3]. In addition, uncorrected refractive errors are a leading cause of moderate to severe vision impairment and the second most common cause of blindness among children worldwide [4]. Consequently, children rely on their vision for acquiring the information necessary for learning. Refractive error impacts the vision of children and affects their intellectual development and learning [5]. Uncorrected refractive error can lead to poor academic performance and lost educational and career opportunities, as well as negatively affect their quality of life [6]. Myopia and hyperopia are known for their negative consequences, resulting in myopic degeneration and closed‐angle glaucoma, respectively [1].

Refractive error is reportedly common among school‐aged children [7]. Nearly 12 million of these children are visually impaired due to uncorrected refractive error [8]. Several studies have been conducted to assess the prevalence of refractive error among schoolchildren. While the studies have consistently shown the presence of this problem among school children, variations exist from what has been reported intra‐ and intercontinentally [9]. Similarly, the prevalence of refractive error varies with age, gender, and geographical region [1, 10].

The World Health Organization recommends that school children undergo visual screening and the correction of any existing refractive error [9, 11]. This emphasized the need for early detection and correction. According to Basnet et al. [12], early detection and correction can help boost the confidence of a child, academic performance, and general well‐being. Nevertheless, the majority of the people who do not have access to refractive error services live in developing countries such as Malawi [13]. Malawi, due to the constrained healthcare system, has many underserved populations, including children [14, 15]. The country, like most developing countries, lacks structured pre‐enrollment and regular school eye health programs; as such, schoolchildren are vulnerable to visually impairing ocular conditions [16].

Previous studies have reported different prevalence rates of refractive errors in sub‐Saharan Africa. For instance, a study in Nigeria reported the prevalence of uncorrected refractive at 11.6% [17], while the prevalence of refractive error among school children in Iran was reported as low as 3.8% [18]. Similarly, a study conducted among school children enrolled in primary school in Ntcheu District, Central Malawi, observed the prevalence of refractive error to be 2.4% [16]. This clearly shows geographical differences in the prevalence of refractive error among studies across different countries, which in part could also reflect on the socioeconomic context of the study. However, to the best of our knowledge, data on the prevalence of refractive error among school children in Northern Malawi remain scant. Therefore, this study was aimed at determining the prevalence of refractive error among school‐going children in five primary schools in Mzuzu City, Northern Malawi. Understanding the prevalence and occurrence of refractive error is vital for eye care planning, policy making, and equitable access to pediatric eye care in Mzuzu City.

## 2. Methods

### 2.1. Research Design

This was a cross‐sectional school‐based study conducted among school‐going children in Mzuzu City. The study was conducted from the 15^th^ of January to the 3^rd^ of February, 2024.

### 2.2. Study Population and Setting

The study population consisted of school‐going children enrolled in three primary schools, namely, Viyere, Chibanji North, and Area 1 B primary schools. Mzuzu City is one of the three cities in Malawi. It is also the only city in the northern region of Malawi. The city has a population of approximately 221,272, of which 98,177 are children. The city had a total of 60,150 primary school enrollments as of 2023, out of which 444 were primary school dropouts. The selected schools had a combined population of 4636 learners, with Area 1 B contributing 1700 learners, Chibanja North contributing 1181 learners, and Viyere Primary School contributing 1755 learners, respectively, and all schools had classes ranging from standards 1 to 8.

### 2.3. Study Sampling Technique and Sample Size

#### 2.3.1. Sample Size

The sample size for this study was determined as 1067 using the Cochran formula *n* *p* (1‐p)/*Z* ^2^/*e* ^2^; however, accounting for nonresponse, the sample population was increased to 1173. The study employed a multistage sampling technique [19], which entailed clustering, proportioning, simple random selection, and a systematic sampling technique to select the participants. The technique involved the random selection of three wards from the list of wards in Mzuzu City. Furthermore, there was the clustering of schools in wards to ensure representativeness of the schools in selected clusters. Then, out of the listed schools, there was a random selection of schools in each cluster. Thereafter, we used a proportion of the study participants from each school to ensure equal representation in terms of percentages based on their level, e.g., grades 1–8. Finally, we selected participants at regular intervals using a systematic sampling method within the grades.

#### 2.3.2. Inclusion and Exclusion Criteria

The study included all the registered primary school children in the three selected schools whose parents consented to the study. Those students who at the time of the examination were absent from school or were sick and/or on medications that can cause fainting were excluded from the study. Furthermore, students who were screened and found to have ocular pathologies such as retinal detachment, cataracts, uveitis, glaucoma, bacterial, viral conjunctivitis, and/or strabismus were excluded from the study to avoid the confounding effect of the conditions on the main outcome. Also, pupils who at the time of this study were diabetic, had a history of autism, and/or had neurological problems were excluded from this study [20].

#### 2.3.3. Examination and Procedures

On the day of examination, the sociodemographic information of the participants (age, gender, and class) was obtained from the participants first. Further details on ocular and medical history were obtained through their health passports. The eye examinations performed included visual acuity measured using Snellen’s visual acuity charts at 6 m. Next, objective refraction was measured using a retinoscope (Keeler R20 ^⟶^ retinoscope), and anterior eye examp was performed using a slitlamp, while intraocular pressure measurement was measured using an iCare tonometer. Cycloplegia was achieved using 1% cyclopentolate. Also, dilated fundus examination was done by using a Volk lens (90D).

The participants were refracted objectively using a retinoscope under dim illumination, using 2 drops of cyclopentolate for cycloplegia instilled 2 min apart. We waited 15 min after cyclopentolate instillation before commencing retinoscope refraction. During the refraction, the participants were given a target to fixate upon, which was the first letter on a Snellen chart 6 m away. Myopia was defined as ≥ −0.5 diopters [21], while hyperopia was ≥ +1.00 diopters [22]. On the other hand, astigmatism was regarded as 0.50 Diopter cylinder [23].

#### 2.3.4. Data Analysis

The data were entered from hard copy to Excel, then SPSS Version 26 for statistical analysis. We used tables to visually illustrate the data. For descriptive statistics, we used frequencies and percentages. Moreover, to compare means between two groups, we used the independent *t*‐test. For bivariate analysis, we employed the chi‐square test to determine associations. Furthermore, we conducted a binary logistic regression to ascertain the effect of the independent variable on the dependent variable. The value of *p* < 0.05 was statistically significant.

#### 2.3.5. Ethics

The ethical clearance for this study was obtained from the Faculty of Health Sciences, Research Ethics Committee of Mzuzu University (FOHS/REC/23/291). Permission to conduct this study was also obtained from the district education manager and the head teachers of the primary schools. Written informed consent was obtained from all the study participants and their parents or carers. Assent was obtained from children less than 18 years old. To ensure that no harm was done to any of the study participants, the data collection adhered to the Declaration of Helsinki.

## 3. Results

### 3.1. Demographic Characteristics of Study Participants

A total of 1173 participants enrolled in three primary schools were recruited for the study, representing a 100% response rate. Among the study population, males and females contributed 586 (49.96%) and 587 (50.04%), respectively (Table 1). The mean age of the participants was 10.22 ± 2.264 years, ranging from 6 to 15 years. The median age was 10. The mean age of males and females was 10.36 ± 2.374 years and 10.27 ± 2.356 years, respectively, with a mean difference of 0.93 ± 0.138 years (*p* > 0.05). Approximately 33 percent (39.3%) of the study participants belonged to the children age group, while 712 (60.7%) comprised adolescents (Table 1).

**TABLE 1:** Demographic characteristics of study participants.

Variable	Category	Frequency	Percent (%)
Age	Children Adolescent	461 712	39.3 60.7
Level of primary	Junior Senior	527 646	44.9 55.1
Gender	Male Female	586 587	50.0 50.0
History of spectacle use	Yes No	14 1159	1.2% 1.2
History of LEE	Yes No	16 1157	1.4 98.6

### 3.2. Distribution of Refractive Error

Out of the 1173 pupils who participated in this study, 649 (55.3%, CI = 0.5252.0.5828) had refractive error, with the refractive error powers ranging from −14.00 to +4.25 D. Hyperopia was the most prevalent type of refractive error, occurring in 610 (52.0%) of the study participants. Myopia was present in 40 (3.4%) while astigmatism was present in 18 (1.5%) subjects (Table 2).

**TABLE 2:** Prevalence of refractive error.

Variables	Frequency (*N*)	(%)
Hyperopia	610	52.0
Myopia	40	3.4
Astigmatism	18	1.5
Emmetropia	505	43.1
Total	1173	100

### 3.3. Factors Assocciated With Prevalence of Refractive Error

As depicted in Table 3, the study indicated that age was associated with refractive errors. A chi‐square test of independence showed that there was a statistically significant association between the prevalence of refractive error and age (*p* = 0.002). The study also found that there was a significant association between the prevalence of refractive error and the level of study (*p* < 0.005). The association between the prevalence of refractive error and the history of the use of spectacles was not statistically significant (*p* < 0.079). However, the history of LEE (*p* = 0.036) was statistically significant (Table 2).

**TABLE 3:** Factors associated with the prevalence of refractive error.

Variable	Emmetrope (%)	RE *n* (%)	*p* value
Sex			0.749
Male	264 (45.1%)	322 (54.9%)	
Female	259 (44.1%)	328 (55.9%)	
Age			0.002^∗^
Children	231 (50.1%)	230 (49.9%)	
Adolescents	292 (41.0%)	420 (59.0%)	
Level of primary			0.005^∗^
Junior primary	259 (49.1%)	268 (50.9%)	
Senior primary	264 (40.9%)	382 (59.1%)	
History of eye spectacles			0.079
Yes	3 (21.4%)	11 (78.6%)	
No	520 (44.9%)	639 (55.1%)	
History of LEE			0.036^∗^
Yes	3 (18.8%)	13 (81.3%)	
No	520 (44.9%)	637 (55.1%)	

*Note:* *p* value^∗^ significant at *p* < 0.05.

### 3.4. Factors Influencing the Prevalence of Refractive Error

As shown in Table 4, a binary logistic regression analysis was conducted to ascertain the effect of age and sex on the dependent variable. The model was statistically significant (*p* = 0.013). The Hosmer and Lemeshow test indicated that the model was a good fit to the data (*x* ^2^ (3) = 5.682 and *p* = 0.128). The Nagelkerke *R* square was 0.016, which means that 1.6% variation in the dependent variable was explained by the independent variables. Overall, the model correctly classified 56.5% of the cases. Accordingly, adolescents had 1.445 times higher odds of having refractive errors compared with younger children (95% CI = 1.141.1.829 and *p* = 0.002).

**TABLE 4:** Factors influencing the prevalence of refractive error.

Variable	OR	95% CI	*p* value
Sex (female)	1.039	0.825.1.309	0.746
Age (adolescents)	1.445	1.141.1.829	0.002
Constant	0.977		0.831

## 4. Discussion

Vision plays a critical role in a child’s learning and educational progress [24]. Other skills acquired during childhood, such as language, hand‐eye coordination, and the interpretation of facial expressions, can also be affected by a child’s vision. As such, several studies, including the present study, have been conducted to determine the prevalence of refractive error among school children in different geographical regions [24–26]. This study observed a high prevalence of refractive error, with hyperopia being the most prevalent condition observed among the schoolchildren. This is in contrast to previous studies which found that myopia was the most common refractive error [21, 24]. Evidence suggests that the prevalence of uncorrected refractive errors varies widely [27].

This study revealed a prevalence rate of refractive error higher than what was reported in other studies conducted among schoolchildren in Nigeria (9.7%) [28] and Ethiopia (4.9%) [29]. Nevertheless, this is closer to the prevalence of refractive error reported in previous studies conducted in Saudi Arabia and India [30], where the prevalence rates of 34.9% and 29.5% were reported, respectively. The variations observed in the studies could be attributed to the differences in the study population, the sample size, and the method of refractive error classification. Unlike the study by Ezinne and Mashige [28], where the prevalence of refractive error was defined as visual acuity less than 6/12, the current study defined refractive error based on cycloplegic retinoscopic findings, which is helpful to unravel latent hyperopia. The high prevalence of refractive error in the study echoes the need to prioritize regular school‐based vision screenings to aid in early detection and correction of refractive error [31]. Failure to do this may result in poor academic performance and even low literacy rate in the country. The children with uncorrected refractive errors in this study were sent to a district hospital for possible correction with spectacles.

Many studies have reported the prevalence of hyperopia to be higher among children living in Africa compared with other types of refractive errors, which can be explained by the comfortable outdoor temperatures which facilitate outdoor activities among these children [32]. In the current study, the prevalence of hyperopia, myopia, and astigmatism was determined as 30.8%, 3.2%, and 1.5%, respectively. This differs from the prevalence of 5.4% and 4.3% reported for hyperopia and myopia in Iran [33]. Contrary to our study findings, myopia was reported to have a higher prevalence (41.8%) among school children in Saudi Arabia [34]. Similarly, the current study findings differ from the reports by Asare and Morjaria [35] and Ajite, Osadare, and Ajite [36], which showed myopia and astigmatism as the most common refractive errors among school children in Ghana (46.7%) and Nigeria (36.2%), respectively. The differences in the current study from those in Ghana could be due to the use of cycloplegia in the determination of the prevalence of refractive error. In the absence of cycloplegia, more myopia and pseudomyopia are often observed; however, with the use of cycloplegia, the maximum hyperopia and latent hyperopia are revealed.

The study observed that despite increasing age, more of the participants were hyperopic. However, there was a progressive increase in the prevalence of myopia. This is similar to the findings in Nigeria, where the myopia prevalence was highest in people between 12 and 15 years old [20]. This supports the work of previous researchers who noted a significant influence of age on the distribution of refractive errors, especially myopia. However, contrary to our study findings, myopia was reported to have a higher prevalence among schoolchildren in Saudi Arabia [34]. The differences show a contrasting distribution in the prevalence of refractive error, which could be due to the different populations involved in the study.

Varying results have been reported on the influence of gender on refractive error by different researchers. In the current study, gender was not a statistically significant predictor of refractive error; however, the prevalence of refractive error was higher compared with their female counterparts. Similarly, gender was also found not to influence the prevalence of refractive error among school children in Abuja, Nigeria [37]. This contradicts the report of higher refractive error prevalence observed in females than in males in Ethiopia [29]. The results of our study can be attributed to the equal number of male and female participants in the study. The differences among studies can be attributed to the different geographical locations among studies.

The current study shows that despite the high prevalence of refractive error, lesser number of the participants who required prescription spectacles had ever worn or was wearing spectacles, thus leaving the condition to be both undetected and not managed. This agrees with the results observed in Onitsha, Nigeria, where 2.0% of the student population with refractive error wore spectacles [28]. The cost of purchasing prescription spectacles remains a challenge for many in Africa due to the high poverty rate and its opportunity cost. However, ready‐made spectacles remain a plausible alternative to reduce the cost of spectacle supply in poor regions like Malawi [38]. Early detection and management of refractive error are critical for the prevention of avoidable causes of blindness. It also promotes better academic performance, as vision is critical to learning.

## 5. Limitations

This is the first study to assess the prevalence of refractive error among school children in Mzuzu City. The use of cycloplegia also meant that latent refractive error was made manifest as such ensuring that the maximum refractive error prevalence was captured. Nonetheless, the study was conducted in an urban setting and, therefore, is not representative of the actual distribution of the prevalence of refractive errors in both rural and urban settings within Mzuzu and Malawi at large. Moreover, the design effect was not considered in the sample size calculation, which could bias the estimation. In addition, this study used *a* +1.00 diopter cutoff, which may explain the unusually high prevalence of hyperopia. Again, the study did not break down the levels of myopia and astigmatism including unaided visual acuity. Besides, the study finding could be affected by emmetropization. Furthermore, the participants of the study were drawn only from public schools within Mzuzu; as such, the results may not be generalized to children enrolled in such schools.

## 6. Recommendations

The high population of students who have refractive errors, without having undergone any form of screening, points to a need for greater efforts at establishing a sustainable school eye health program within the region. Therefore, this study recommends that there should be an established school eye health program to ensure that every child has an opportunity for visual screening. Furthermore, there is a need to reduce the cost of spectacles through ensuring the provision of readymade spectacles to indigent pupils whose parents may not afford regular spectacles. There is a need to conduct further studies to evaluate the experiences of school‐going children with visual impairment to fully elucidate this phenomenon. In addition, there is a need for more studies related to the prevalence of refractive error and binocular vision status among school‐going children.

## 7. Conclusion

The study found that a large proportion of students had uncorrected refractive error, with a prevalence rate of 35.5%. The distribution of hyperopia, myopia, and astigmatism was 30.8%, 3.2%, and 1.5%, respectively. Less than 10% of the entire population had ever undergone any form of eye screening, irrespective of their age and refractive error status. Among those with a refractive error, less than 2% wore corrective spectacles. The study highlights the high unmet needs for refractive error services, including spectacle coverage, among a vulnerable population group in a low‐resource setting. This points to a significant public health concern that requires intervention.

## Funding

The authors have nothing to report.

## Ethics Statement

The study was ethically approved by an institution review board.

## Conflicts of Interest

The authors declare no conflicts of interest.

## Data Availability

The data used to support the findings of this study are available from the corresponding author upon reasonable request.
